# Discovering novel SNPs that are correlated with patient outcome in a Singaporean cancer patient cohort treated with gemcitabine-based chemotherapy

**DOI:** 10.1186/s12885-018-4471-x

**Published:** 2018-05-11

**Authors:** Vachiranee Limviphuvadh, Chee Seng Tan, Fumikazu Konishi, Piroon Jenjaroenpun, Joy Shengnan Xiang, Yuliya Kremenska, Yar Soe Mu, Nicholas Syn, Soo Chin Lee, Ross A. Soo, Frank Eisenhaber, Sebastian Maurer-Stroh, Wei Peng Yong

**Affiliations:** 1 Bioinformatics Institute (BII), Agency for Science, Technology and Research (A*STAR), 30 Biopolis Street, #07-01 Matrix, Singapore, 138671 Singapore; 2 Department of Haematology-Oncology, National University Health System, 5 Lower Kent Ridge Road, Singapore, 119074 Singapore; 3 Education Academy of Computational Life Sciences, Tokyo Institute of Technology, Tokyo, Japan; 4 Yong Loo Lin School of Medicine, National University of Singapore, Singapore, Singapore; 5 Cancer Science Institute of Singapore, National University of Singapore, Singapore, Singapore; 6 Department of Biological Sciences, National University of Singapore (NUS), 14 Science Drive 4, Singapore, 117543 Singapore; 7 School of Computer Engineering (SCE), Nanyang Technological University (NTU), 50 Nanyang Drive, Singapore, 637553 Singapore

**Keywords:** Gemcitabine, NSCLC, Pharmacogenetics, SNPs, Patient outcome

## Abstract

**Background:**

Single Nucleotide Polymorphisms (SNPs) can influence patient outcome such as drug response and toxicity after drug intervention. The purpose of this study is to develop a systematic pathway approach to accurately and efficiently predict novel non-synonymous SNPs (nsSNPs) that could be causative to gemcitabine-based chemotherapy treatment outcome in Singaporean non-small cell lung cancer (NSCLC) patients.

**Methods:**

Using a pathway approach that incorporates comprehensive protein-protein interaction data to systematically extend the gemcitabine pharmacologic pathway, we identified 77 related nsSNPs, common in the Singaporean population. After that, we used five computational criteria to prioritize the SNPs based on their importance for protein function. We specifically selected and screened six candidate SNPs in a patient cohort with NSCLC treated with gemcitabine-based chemotherapy.

**Result:**

We performed survival analysis followed by hematologic toxicity analyses and found that three of six candidate SNPs are significantly correlated with the patient outcome (*P* < 0.05) i.e. ABCG2 Q141K (rs2231142), SLC29A3 S158F (rs780668) and POLR2A N764K (rs2228130).

**Conclusions:**

Our computational SNP candidate enrichment workflow approach was able to identify several high confidence biomarkers predictive for personalized drug treatment outcome while providing a rationale for a molecular mechanism of the SNP effect.

**Trial registration:**

NCT00695994. Registered 10 June, 2008 ‘retrospectively registered’.

**Electronic supplementary material:**

The online version of this article (10.1186/s12885-018-4471-x) contains supplementary material, which is available to authorized users.

## Background

Gemcitabine (2′-2′ difluorodeoxycytidine) is a deoxycytidine analogue with antitumor activity against a variety of solid tumors such as non-small cell lung cancer (NSCLC), breast cancer [1] and pancreatic cancer [2]. Gemcitabine requires phosphorylation to mono-, di-, and triphosphates (dFdCTP) to be active. This mechanism results in a unique pattern of self-potentiation of the drug and when this drug is incorporated into the DNA during replication, it causes chain termination. Gemcitabine also has multiple intracellular targets. Up- or downregulation of these targets may confer resistance to this drug.

Wider availability and lower costs of genome and expression profile sequencing made application of those techniques in clinical practice feasible; thus, the scientific question of how patient-specific mutations and chromosomal aberrations influence personal clinical outcomes via biomolecular mechanisms has become acute [3, 4]. For example, pharmacogenetics studies published in the last decades have provided evidence that Single Nucleotide Polymorphisms (SNPs) can causally influence patient outcome such as drug response and toxicity after drug intervention [5]. Most SNPs associated with patient outcome have been found in genes involved in the drug pharmacology i.e. affecting drug transport, metabolism and/or activity with drugs. Soo et al. tested 26 SNPs from nine genes that are already known to be directly associated with gemcitabine transport, metabolism and activity [6]. They found several SNPs that were associated with patient outcome in Singaporean NSCLC patients treated with gemcitabine [6]. However, a systematic approach to investigate the relationship between gene variants and patient outcome is still lacking. Therefore, the purpose of this study is to develop a systematic pathway approach to accurately and efficiently predict novel non-synonymous SNPs (nsSNPs) that could be causative to gemcitabine-based chemotherapy treatment outcome in Singaporean NSCLC patients. After detailed SNP analysis, we prioritized the SNPs based on their importance in protein function and molecular mechanism. From the top-ranking SNPs, we specifically selected six final candidate SNPs for clinical validation. We genotyped these six SNPs in a Singaporean patient cohort and have found that three out of the six SNPs correlated with patient outcome.

## Methods

### Pyrimidine metabolism as a starting pathway to find more genes in the gemcitabine pharmacologic pathway

Pyrimidine metabolism is known to be critical in the pharmacologic pathway of gemcitabine, which is a pyrimidine analogue. Therefore, we used the pyrimidine metabolism pathway in KEGG (hsa00240; http://www.genome.jp/kegg-bin/show_pathway?org_name=hsa&mapno=00240&mapscale=&show_fdescription=show) [7] which contains 100 genes as a starting point. In addition, we also did extensive literature search to find more genes that are directly associated with gemcitabine transport [6, 8, 9]. Apart from literature review, information from PharmGKB (http://www.pharmgkb.org/) has also been referenced. As a result, six membrane transporters implicated in the uptake of gemcitabine i.e. SLC28A1 (Entrez GeneID:9154), SLC28A2 (Entrez GeneID:9153), SLC28A3 (Entrez GeneID:64078), SLC29A1 (Entrez GeneID:2030), SLC29A2 (Entrez GeneID:3177), SLC29A3 (Entrez GeneID:55315) and three transporters implicated in the efflux of gemcitabine i.e. ABCC5 (Entrez GeneID:10057), ABCC10 (Entrez GeneID:89845) and ABCG2 (Entrez GeneID:9429) were added to the pathway. In total, 109 genes (the 100 genes in pyrimidine metabolism and the 9 membrane transporter genes) were used as starting proteins to find more interaction partners by using our in-house comprehensive protein-protein interaction (PPI) data.

### Adding more potentially related proteins to the pathway using comprehensive PPI data

We used our in-house comprehensive PPI data to find additional proteins that could be related to the gemcitabine pharmacologic pathway. Comprehensive PPI data was consolidated by integrating experimentally-validated PPIs from nine databases, i.e. BIND [10], BioGRID [11], IntAct [12], DIP [13], MINT [14], MPact [15], HPRD [16], GNP (http://genomenetwork.nig.ac.jp/index_e.html) and MPPI [17], to provide unique PPIs together with the accumulation of evidence such as experimental type and PubMed IDs. The method we used to integrate multiple databases is provided in detail in this website i.e. http://ipid.bii.a-star.edu.sg/annie/home.do#ui-tabs-1. The final interaction set contains 1,148,484 unique PPIs including 227,731 human PPIs. We used only human PPIs in this study. We extended the pyrimidine metabolism in KEGG (hsa00240) using the conservative requirement that the new protein must have been reported to interact with at least two out of the 100 proteins that are already in the pathway. After that, we collected nsSNPs from NCBI/dbSNPs build 136 [18] that are linked to these human genes using the NCBI E-utilities tool [19] with search terms “missense”, “nonsense” or “frameshift”. Information from databases “ensembl_mart_66” and “homo_sapiens_variation_66_37” [20] was then used to annotate each of the nsSNPs retrieved from E-utilities i.e. Ensembl’s genotype, Ensembl’s transcription ID, Ensembl’s consequence type, NCBI’s consequence type, HGVS genomic, HGVS coding, HGVS protein, PolyPhen-2 and SIFT prediction for reference. A script was written in python and was run on 1st March 2012.

### Finding common SNPs in the Singaporean population

We used allele frequency information from the Singapore Genome Variation Project (SGVP) [21] to find common SNPs in the Singaporean population among the retrieved nsSNPs. The SGVP provides a publicly available resource of 1.6 million SNPs genotyped in 268 individuals from the Chinese, Malay, and Indian ethnicities in the Singaporean population. In this study, a common SNP is defined as one with a minor allele frequency (MAF) of ≥5% in at least one out of three ethnic groups i.e. Chinese, Malays or Indians.

### Five criteria to filter candidate SNPs

After common SNPs are selected from the retrieved SNPs, five criteria were used to narrow down the common SNPs to select only those that are likely to affect protein function i.e.

4.1) The SNP’s MAF from SGVP is specifically higher in the Singaporean Chinese compared to the Singaporean Malay or the Singaporean Indian ethnicity since about 87% of our patients are Singaporean Chinese (refer to Table 1).
4.2) Result of PolyPhen-2 [22] prediction is “possibly damaging” or “probably damaging” by using rsID of each SNP as input. We used the batch query option of PolyPhen-2 with HumDiv classifier model and genome assembly GRCh37/hg19. For those SNPs that could not retrieve result from the batch query option, we input rsID one by one to the PolyPhen-2 website (http://genetics.bwh.harvard.edu/pph2/) to retrieve the result.
4.3) Result of SIFT [23] prediction is “Affect protein function”. SIFT results were first retrieved using “SIFT dbSNP batch tool” which was run on 21 March 2012 to pre-screen the results. After that, orthologue sequences (select only “1:1 orthologs”) were retrieved from either OMA browser [24] or Orthologue search against NCBI Non-redundant protein set on ANNOTATOR [25] and were used to create a multiple sequence alignment with MAFFT (L-INS-I settings) [26]. We deleted those sequences that have large gaps using Jalview [27].
4.4) A SNP is located in the functional domain of a protein. We used the amino acid sequence of the gene that the SNP is located in as input to do “Prim-Seq-An w/Pfam” analysis in ANNOTATOR [25] using default settings which include HMMER against many protein domain databases e.g. SMART, Pfam to retrieve functional domain information of the protein. Later, we annotated whether a SNP is located in any functional domain of the corresponding protein or not.
4.5) Average free energy change (ddG, kcal/mol) of the protein by the SNP as predicted by FoldX from 5 runs [28] is significant i.e. more than 0.5 kcal/mol or less than − 0.5 kcal/mol. The menu option “Mutate residue” in the FoldX plugin for YASARA [29] was used to predict free energy changes of the protein when the wild-type amino acid is mutated to another amino acid to predict the effect of SNPs on protein structure. The structure of the protein associated with the SNP of interest was energy minimized using the “RepairPDB” function in FoldX before mutating the residue from wild-type amino acid to the SNP’s amino acid and calculating stability change. To perform this analysis, a 3D protein structure or homology model is needed, so a template or crystal structure that contains the SNP’s region is retrieved by using either “NCBI-BLAST” of the protein sequence against PDB (E-Value cutoff 0.001 with BLOSUM62 matrix) or HHPRED against PDB (E-Value cutoff 0.001) on ANNOTATOR [25]. If there is a crystal structure available where the SNP is located, we use the crystal structure as an input to FoldX. For SNPs in proteins without crystal structures but found to have appropriate homologous template structures, we model the structure by homology modeling using MODELLER [30] with loop refinement.

**Table 1:** Characteristics of patients who were treated with gemcitabine-based chemotherapy

Characteristics at diagnosis	NSCLC patients (*n* = 92)^a^
Ethnicity	
Chinese	80
Malay	9
Indian	0
Others	2
No data	1
Gender	
Male	67
Female	24
No data	1
Stage of Cancer	
Stage III	14
Stage IV	77
No data	1
Performance Status (ECOG)	
0	58
1	33
No data	1

^a^Could not retrieve any data from one patient and there is another patient who had no survival data

Finally, after consideration of the five criteria in each of the 77 SNPs, we selected only the top-ranking candidate SNPs for genotyping in the Singaporean patient cohort with known clinical trial data.

### Study population

The Singapore National Healthcare Group Domain Specific Review Board reviewed and approved the study. All the patients provided written informed consent before study entry. The study was conducted in accordance to Good Clinical Practice guidelines. A total of 92 non-small cell lung cancer (NSCLC) patients were recruited for the study and were analysed. All the patients received their treatments in the Department of Haematology-Oncology at National University Hospital of Singapore. Patients with not more than two lines of prior systemic chemotherapy were recruited to receive gemcitabine (750-1000 mg/m^2^ on day 1 and day 8) and carboplatin (AUC 5 mg/ml on day 1) every 3 weeks. Radiographic assessments were done to evaluate tumor response every two cycles according to RECIST criteria. Safety assessments were performed at every cycle including weekly full blood counts to monitor haematological toxicities. Demographic profiles of the patients are summarized in Table 1. We could not retrieve any data from one NSCLC patient and there is another stage IV NSCLC Chinese, male with ECOG = 0 patient who had no survival data as well. So in total, 90 NSCLC patients were available for survival and toxicity analysis.

### Blood collection and genomic DNA extraction

A total of 8 ml peripheral blood was obtained from each patient. The blood was drawn into heparinized vacutainer tubes (Becton Dickinson) and mononuclear cells isolated by Ficoll-Hypaque density gradient centrifugation according to manufacturer’s instructions (GE Healthcare, Chalfont St Giles, United Kingdom). The DNA in turn was extracted from the mononuclear cells using the Puregene DNA purification kit (Gentra Systems, Minneapolis, MN).

### PCR (polymerase chain reaction) and pyrosequencing

First, PCR products were immobilized on streptavidin-coated beads and denatured to produce single-stranded products. Pyrosequencing was performed using the PyroMark Gold Q24 reagent and the PyroMark Q24 system (Qiagen), according to the manufacturer’s protocol. Primers for pyrosequencing were designed with the PyroMark Assay Design Software 2.0. Primers, including biotin-labelled and sequencing primers are represented in Additional file 1: Table S1. Sequencing analysis was performed using PyroMark Q24 version 2.0.6 software in the allele quantification analysis (QA) mode.

### Statistical analysis to find correlation between the candidate SNPs and patient outcome

Kaplan-Meier methods and log-rank test were used to analyse results for overall survival and progression-free survival in the NSCLC patient cohort. Grade 3 or 4 haematological toxicities and its association with gene variants were analysed using Chi-squared test. All statistical analyses were two-sided and the SPSS software version 16.0 was used. *P* value of less than 0.05 were considered to indicate nominal statistical significance. Predictor variables – including gender, age, stage and ECOG), and the 6 SNPs – were initially correlated with categorical outcomes (grade 3/4 neutropenia and thrombocytopenia) using the chi-squared test, and with time-to-event outcomes (overall and progression free survival) using the log-rank test in univariate fashion. Next, clinical variables and SNPs which were found to be significant in the univariate analyses were included in multivariate Cox or logistic regression to obtain adjusted *p* values and effect sizes.

## Results

### 5046 nsSNPs were found to be linked to the 178 genes in the gemcitabine pharmacologic pathway

**Fig. 1:**
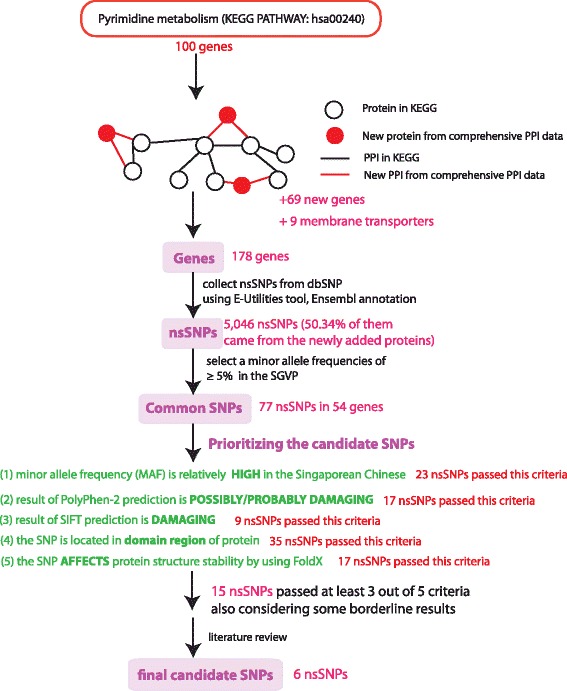
Overall workflow and summary of results in each step. The pyrimidine metabolism (KEGG PATHWAY: hsa00240, 100 genes) has been chosen as a starting point and then using comprehensive PPI to extend the pathway to add more proteins that could be potentially related to the pathway in which 69 new proteins can be added. We also added 9 membrane transporters that have been known to be associated with the gemcitabine pharmacologic pathway. 5046 nsSNPs are found to be linked to the 178 genes (100 together with the new 69 and the 9 transporters’ genes). 77 of them are found to be common in Singaporean population. After that, five criteria have been used to prioritize the common SNPs. We did detailed SNP analysis for 15 common nsSNPs that passed at least 3 out of 5 criteria and some borderline SNPs. Finally, after thorough literature review, we selected six SNPs to be genotyped in the NSCLC Singaporean patient cohort. PPI: Protein-protein interaction, SGVP: Singaporean Genome Variation Project

The overall workflow and result in each step are described in Fig. 1. We used 100 proteins in the human pyrimidine metabolism metabolic pathway (KEGG:hsa00240) as a starting point and then used our in-house comprehensive PPI data which comprise of unique 227,731 human PPIs integrated from nine public databases (detail in Method section) to extend the pathway by using a conservative requirement that the new protein needs to connect to at least two other out of the 100 proteins that are already in the pyrimidine pathway. By using this criterion, we found an additional 69 proteins from the comprehensive PPI data that can be connected to the pathway. Therefore, 169 genes (100 genes in the pyrimidine metabolism and an additional 69 new genes) together with the 9 membrane transporters from literature review were used to find nsSNPs that are linked to these genes. By using NCBI E-utilities, 5046 nsSNPs were found to be linked to all 178 genes from dbSNPs. 2540 of these nsSNPs (50.34%) came from the newly added proteins. Next, using allele frequency data from SGVP [21], we found that only 77 (in 54 genes) of the retrieved nsSNPs have MAF with more than or equal to 5% in at least one ethnicity in the Singaporean population. We called these 77 nsSNPs as common SNPs in this study. These common SNPs contain 73 missense, 3 nonsense and 1 frameshift mutations. Detailed information of all common nsSNPs are described in Additional file 1: Table S2. Among the 77 common SNPs, eight were found to be previously tested in the Singaporean patient cohort in NSCLC [6] (please refer “*” after rsIDs in Additional file 1: Table S2). Out of the eight, three of them were proven to be associated with patient outcome i.e. SLC28A1 D521N (rs2242046), SLC28A2 P22L (rs11854484) and SLC28A2 S75R (rs1060896) although these SNPs passed only one or none in our criteria (Additional file 1: Table S2). Later, POLA2 G583R (rs487989) which is one of the eight SNPs and passed two of our criteria was proven to be strongly associated with mortality rate and survival time among Singaporean NSCLC patients treated with gemcitabine [31].

### 15 out of 77 common nsSNPs have significant results in three out of five criteria

We used five criteria (as described in detail in the Method section) to narrow down the common SNPs to select only those that are likely to affect protein function (Fig. 1). The first criterion was to select SNPs that have higher MAF in the Chinese population since the majority of our patients are Singaporean Chinese. 23 out of the 77 nsSNPs were found to match this criterion. The second and third criteria were based on prediction results from PolyPhen-2 and SIFT, respectively, both of which are evolutionary sequence conservation-based approaches. We used the batch-query tool of PolyPhen-2 to parse pre-calculated results of all common nsSNPs. For those SNPs that could not fetch results from the batch-query tool, we used RefSeq amino acid sequence ID of each gene as input to retrieve the result from the PolyPhen-2 website. From the PolyPhen-2 result (i.e. the second criteria), there were 17 common SNPs predicted to be either “probably” or “possibly damaging” (Additional file 1: Table S2). For the SIFT analysis (i.e. the third criteria), we used the “SIFT dbSNP batch tool” to retrieve prediction results for all common nsSNPs and found that only 9 of them were predicted as “Deleterious” with the SIFT score equal to or less than 0.05 (Additional file 1: Table S2). The fourth criterion is to check if the SNPs lie on any functional domain. To do this, we retrieved HMMER results against Pfam and SMART using “Prim-Seq-An w/Pfam” analysis on ANNOTATOR and found that 35 out of the 77 SNPs are located in a functional domain of their proteins. The final criterion was to investigate whether the SNP affects protein structural stability using FoldX. Only 27 common SNPs were in a region with a known structure or high similarity to a known structure which allows us to do homology modeling and FoldX analysis. 17 of them returned significant results from FoldX. Results of the five criteria of all 77 common nsSNPs are described in Additional file 1: Table S2.

**Fig. 2:**
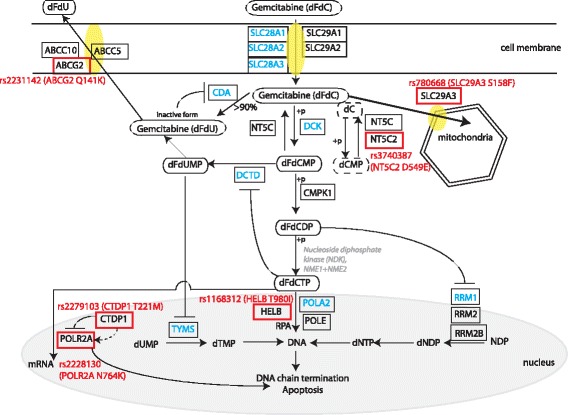
Schematic diagram of gemcitabine pharmacologic pathway. Key genes that are directly involved in the gemcitabine pharmacologic pathway are shown. Genes in blue have been studied or tested with NSCLC patient samples previously in other publications. Other genes are found from our pathway-based approach. Nine membrane transporters that are included in this study are also shown in this diagram i.e. ABCC10, ABCC5, ABCG2, SLC28A1, SLC28A2, SLC28A3, SLC29A1, SLC29A2 and SLC29A3. The six SNPs which belong to six genes (in red box) were selected as final candidate SNPs in our study

Finally, we were able to narrow down the 77 common nsSNPs to 15 which have significant results in at least three out of the five criteria (Additional file 1: Table S2). We also considered some SNPs that retrieved border line results. Lastly, literature review was performed to understand the functional role of the genes associated with the SNPs to further select SNPs that are most likely to affect the gemcitabine pathway. Finally, we identified the following six SNPs, that is, ABCG2 Q141K (c.421C > A, rs2231142), SLC29A3 S158F (c.473C > T, rs780668), HELB T980I (C > T. rs1168312), NT5C2 D549E (c.1647C > T, rs3740387), POLR2A N764K (c.2292C > T, rs2228130) and CTDP1 T221M (c.662C > T, rs2279103) as final candidate SNPs based on the importance of the SNPs for protein function and drug-related molecular mechanisms (Fig. 2). Five out of the six final candidate SNPs passed three out of five criteria and only CTDP1 T221M is selected based on a borderline result of the computational selection criteria because it seemed plausible from our literature study. These SNPs are then genotyped in a Singaporean NSCLC patient cohort with known patient outcome for gemcitabine-based therapy.

### Genotyping of the six final candidate SNPs

A total of 90 NSCLC patients that have survival data available were genotyped for the six final candidate SNPs. Genotype information of 90 samples is shown in Additional file 1: Table S3. Genotype of HELB T980I could not be retrieved from 2 out of 90 patients. Therefore, we used 88 NSCLC patient data to perform survival and toxicity analyses in the next step. We could not find any patient who has the TT genotype of POLR2A N764K.

### ABCG2 Q141K and SLC29A3 S158F are associated with increased survival in NSCLC

Kaplan-Meier analysis was performed to determine any correlation of the six final candidate SNPs with overall survival (OS) and progression free survival (PFS) in the NSCLC patient cohort (*n* = 88). ABCG2 Q141K (c.421 C > A, rs2231142) was found to be associated with increased median PFS. Patients with CA/AA genotype were shown to have longer PFS compared to CC genotype i.e. 9.12 months [95% CI 1.83-16.4 months] vs 5.51 months [95% CI 4.31-6.71 months] respectively, HR 0.51 (95% CI 0.31-0.83), adjusted *P =* 0.007 (Table 2).

**Table 2:** Univariate and multivariate Cox regression analyses of progression-free survival (PFS) and overall survival (OS) of the six final candidate SNPs with clinical parameters in the NSCLC cohort

	Factors/Genotype	Number/	Progression Free Survival (PFS)				Number/	Overall Survival (OS)			
		Median PFS (months)	Univariate analysis		Multivariate analysis		Median OS (months)	Univariate analysis		Multivariate analysis	
			HR (95% CI)	*P* value	HR (95%CI)	*P* value (adjusted)		HR (95% CI)	*P* value	HR (95%CI)	*P* value (adjusted)
Gender	Male	66	0.92 (0.55-1.55)	0.767	–	–	66	0.76 (0.43-1.36)	0.357	–	–
	Female	22					22				
Age	< 62	42	0.81 (0.51-1.28)	0.370	–	–	42	0.85 (0.51-1.40)	0.523	–	–
	≥62	46					46				
Stage	3	19	1.59 (0.91-2.76)	0.100	–	–	19	1.60 (0.84-3.02)	0.151	–	–
	4	69					69				
ECOG	0	27	1.22 (0.73-2.04)	0.451	–	–	27	**1.95 (1.06-3.57)**	**0.031**	**2.13 (1.14-3.97)**	**0.018**
	1	61					61				
ABCG2 Q141K	CC	5.51	**0.48 (0.29-0.78)**	**0.003**	**0.51 (0.31-0.83)**	**0.007**	11.57	**0.52 (0.31-0.89)**	**0.017**	0.61 (0.35-1.06)	0.080
	CA/AA	9.12					20.19				
SLC29A3 S158F	CC	5.08	**0.56 (0.33-0.96)**	**0.034**	0.64 (0.37-1.10)	0.108	8.43	**0.52 (0.30-0.92)**	**0.024**	**0.49 (0.27-0.88)**	**0.017**
	CT/TT	7.84					17.64				
NT5C2 D549E^a^	CC	5.25	0.71 (0.41-1.21)	0.206	–	–	10.49	0.67 (0.38-1.17)	0.157	–	–
	CT/TT	7.57					17.31				
HELB T980I	CC	8.2	1.42 (0.90-2.25)	0.133	–	–	17.05	1.23 (0.75-2.04)	0.412	–	–
	CT/TT	5.74					11.28				
CTDP1 T221M	CC	7.02	1.24 (0.74-2.09)	0.421	–	–	13.35	1.10 (0.62-1.96)	0.741	–	–
	CT/TT	6.56					16.85				
POLR2A N764K	CC	7.02	1.15 (0.55-2.42)	0.711	–	–	13.34	1.14 (0.54-2.40)	0.734	–	–
	CT	7.57					14.03				

^a^Latest dbSNP build annotated this SNP as NT5C2 D549D (synonymous snp)

Data in bold are those that have p < 0.05

SLC29A3 S158F (c.473C > T, rs780668) was found to be associated with increased OS. Patients with CT/TT genotype were shown to have longer median OS compared to CC genotype i.e. 17.64 months [95% CI 10.55-24.73 months] vs 8.43 months [94% CI 1.21-15.64 months], HR 0.49 (95% CI 0.27-0.88), adjusted *P =* 0.017 (Table 2). Association with OS/PFS could not be found in four other variants (Table 2).

### ABCG2 Q141K and POLR2A N764K are correlated with gemcitabine cytotoxicity

The ABCG2 Q141K variant (the CA/AA genotype) was not only associated with improved PFS but was also found to be associated with increased toxicity i.e. higher risk of grade 3 or 4 thrombocytopenia (low platelet count) compared to the wild-type genotype (CC) (70.7% vs 44.7% respectively, HR 3.79 (95% CI 1.42-10.1) adjusted *P* = 0.008) (Table 3). Interestingly, the wild-type CC genotype of POLR2A N764K variant was found to be associated with a higher risk of grade 3 or 4 thrombocytopenia at 61.5% compared to 20.0% of the SNP’s CT genotype, HR 0.18 (95% CI 0.03-0.98), adjusted *P* = 0.048 (Table 3).

**Table 3:** Univariate and multivariate analyses by chi square and logistic regression, respectively, of grade 3 or 4 neutropenia and thrombocytopenia of the six final candidate SNPs with clinical parameters in the NSCLC cohort

	Factors/Genotype	Number/ Percentage of grade 3/4 neutropenia (all cycles)	Grade 3/4 neutropenia				Number/Percentage of grade 3/4 thrombocytopenia (all cycles)	Grade 3/4 thrombocytopenia			
			Univariate analysis		Multivariate analysis			Univariate analysis		Multivariate analysis	
			OR (95%CI)	*P* value	OR (95%CI)	*P* value (adjusted)		OR (95%CI)	*P* value	OR (95%CI)	*P* value (adjusted)
Gender	Male	66	2.74 (0.83-9.04)	0.097	–	–	66	**4.78 (1.46-15.7)**	**0.01**	**4.44 (1.27-15.6)**	**0.020**
	Female	22					22				
Age	< 62	42	1.03 (0.42-2.51)	0.942	–	–	42	0.56 (0.24-1.31)	0.178	**–**	**–**
	≥62	46					46				
Stage	3	19	2.21 (0.78-6.24)	0.136	–	–	19	2.84 (0.99-9.12)	0.052	**–**	**–**
	4	69					69				
ECOG	0	27	0.80 (0.30-2.14)	0.659	–	–	27	0.87 (0.35-2.17)	0.758	**–**	**–**
	1	61					61				
ABCG2 Q141K	CC	59.60%	2.10 (0.84-5.28)	0.113	–	–	44.70%	**2.99 (1.23-7.25)**	**0.015**	**3.79 (1.42-10.1)**	**0.008**
	CA/AA	75.60%					70.70%				
SLC29A3 S158F	CC	50.00%	2.67 (0.99-7.22)	0.054	–	–	45.60%	1.85 (0.70-4.89)	0.109	**–**	**–**
	CT/TT	72.70%					60.60%				
NT5C2 D549E^*^	CC	57.10%	1.76 (0.64-4.84)	0.272	–	–	57.10%	0.98 (0.37-2.65)	0.973	**–**	**–**
	CT/TT	70.20%					56.70%				
HELB T980I	CC	70.00%	0.73 (0.30-1.80)	0.499	–	–	56.00%	1.08 (0.46-2.53)	0.859	**–**	**–**
	CT/TT	63.20%					57.90%				
CTDP1 T221M	CC	66.70%	1.07 (0.38-3.01)	0.090	–	–	54.60%	1.46 (0.54-3.94)	0.457	**–**	**–**
	CT/TT	68.20%					63.60%				
POLR2A N764K	CC CT	70.50 40.00%	0.28 (0.07-1.08)	0.065	–	–	61.50 20.00%	**0.16 (0.03-0.79)**	**0.024**	**0.18 (0.03-0.98)**	**0.048**

Data in bold are those that have p < 0.05

## Discussion

**Fig. 3:**
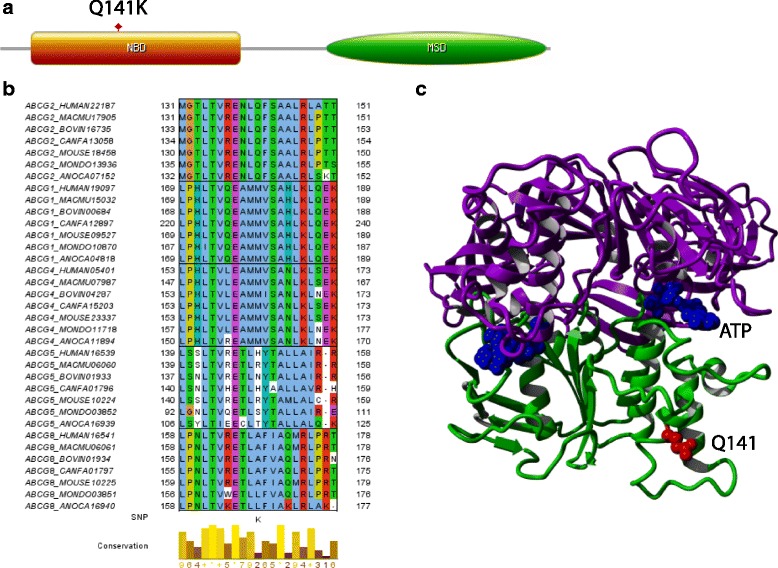
Result of detailed SNP analysis of ABCG2 Q141K. **a** Domain architecture of ABCG2. ABCG2 contains a nucleotide-binding domain (NBD) in the cytoplasmic region and a membrane-spanning domain transmembrane domain (MSD) consisting of 6 putative transmembrane segments. ABCG2 Q141K is located in the NBD. **b** Multiple alignment using all five members in the ABCG subfamily. Orthologs of each member were retrieved from OMA browser (omabrowser.org/). MAFFT with L-INS-i was used to create the multiple alignment. We used seven representative organisms to show conservation of SNP’s region. HUMAN: *H. sapiens*, MACMU: *M. mulatta*, BOVIN: *B. taurus*, CANFA: *C. familiaris*, MOUSE: *M.musculus*, MONDO: *M.domestica*, ANOCA: *A. carolinensis*. **c** Homology model of the nucleotide-binding domain of ABCG2 using the ATP subunit of the maltose transporter from *E.coli* (PDB:1Q12 chain A) [37] as a template is shown in green. ATPs are shown in blue and Q141 is shown in red. Superimposition of the model and chain B of the template (shown in purple) was done to show the homodimer of the region

**Fig. 4:**
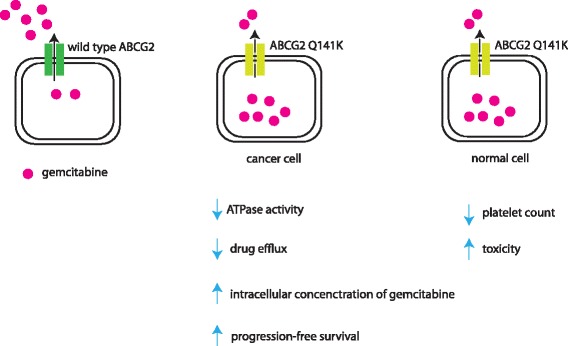
Proposed mechanisms of ABCG2 Q141K SNP and patient outcome. NSCLC patient who is treated with gemcitabine and have ABCG2 Q141K either heterozygous or homozygous allele could increase their survival because of accumulating more gemcitabine inside cancer cells and is thus more effective in killing cancer cells. However, probability of increasing toxicity can occur since other substrates of ABCG2 can be accumulated inside normal/healthy cells and thus cause cell death. These healthy cells can include cells in the bone marrow that produce blood e.g. platelets

In this study, three out of the six candidate SNPs were confirmed to be associated with NSCLC patient outcome i.e. OS, PFS and side effect. To the best of our knowledge, this is the first study showing association of ABCG2 Q141K (rs2231142), SLC29A3 S158F (rs780668) and POLR2A N764K (rs2228130) with NSCLC patient outcome treated with gemcitabine-based chemotherapy. ABCG2 belongs to the ABCG subfamily and ABC transporter superfamily. The ABCG family has five members i.e. ABCG2, ABCG1, ABCG4, ABCG5 and ABCG8. ABCG2 consists of a nucleotide-binding domain (NBD) in the amino terminus followed by six putative transmembrane domains (Fig. 3a). The ABCG2 Q141K SNP is located at the NBD in the cytoplasmic part of the protein. The c.421A allele frequency of ABCG2 Q141K is known as one of the common SNPs in Asian people (about 26-35%) [32]. Moreover, this SNP has been shown to be associated with increased risk of gout [33]. When we created our own detailed multiple sequence alignment using all members in the ABCG family, we found that glutamine in this position is well conserved in ABCG2 orthologs but not in other members in the family, therefore Q141 can be considered as an ABCG2-subfamily specific conserved residue (Fig. 3b). ABCG2 is the only member in this family that is not involved in cholesterol efflux but it mediates the efflux of a wide range of xenobiotics including gemcitabine, using ATP as an energy source [34]. There is in vitro evidence that ABCG2 Q141K decreases efflux activity and increases intracellular gemcitabine levels and it has been known to be associated with impaired ABCG2 activity by lowering protein expression level or decreasing ATPase activity [35]. The study supports the observation that ABCG2 itself plays a role in decreasing intracellular concentration of gemcitabine. Another in vitro study demonstrates significantly worse overall survival for carriers of the ABCG2 421A-allele treated with platinum-based drugs [36]. Mizuarai et al. described that the ATPase activity of the Q141K variant was reduced approximately 1.3-fold compared to the activity of the wild type ABCG2 in polarized LLC-PK1 cell lines, resulting in increased drug accumulation and decreased drug efflux in the variant ABCG2-expressing cells [36]. According to BLAST against PDB, a crystal structure of Malk, the ATP subunit of the maltose transporter from *E.coli* (PDB:1Q12 chain A) [37] was the top hit with a E-value of 2.0E-17. We used this template to do homology modeling of the NBD region (position 41-299) of ABCG2 using MODELLER. The ABCG2 model is shown in Fig. 3c. We used this model to calculate the stability change upon mutation by FoldX and found the average free energy changes (ddG) when mutating Q to K at position 141 of ABCG2 to be 1.93 kcal/mol with a standard deviation (SD) of 0.10 kcal/mol. This suggests that the SNP has a destabilizing effect on the protein structure which is in agreement with a recent finding that Q141 causes instability in the NBD [38]. The SNP is located in the loop region which is relatively near the ATP binding site of the dimer and changing the neutral side-chain glutamine to positively-charged side-chain lysine may affect the scaffold of the neighboring ATP binding site formed by the homodimer (Fig. 3c). Therefore, it can be proposed that if a patient has this variant and is treated with gemcitabine, efflux of gemcitabine can be diminished resulting in an increase in the intracellular concentration of gemcitabine in cancer cells and it is thus more effective at killing cancer cells. However, since normal cells also have this SNP which causes accumulation of the drug and other substrates exported by this protein, this SNP is also linked to increased toxicity in normal cells (Fig. 4).

**Fig. 5:**
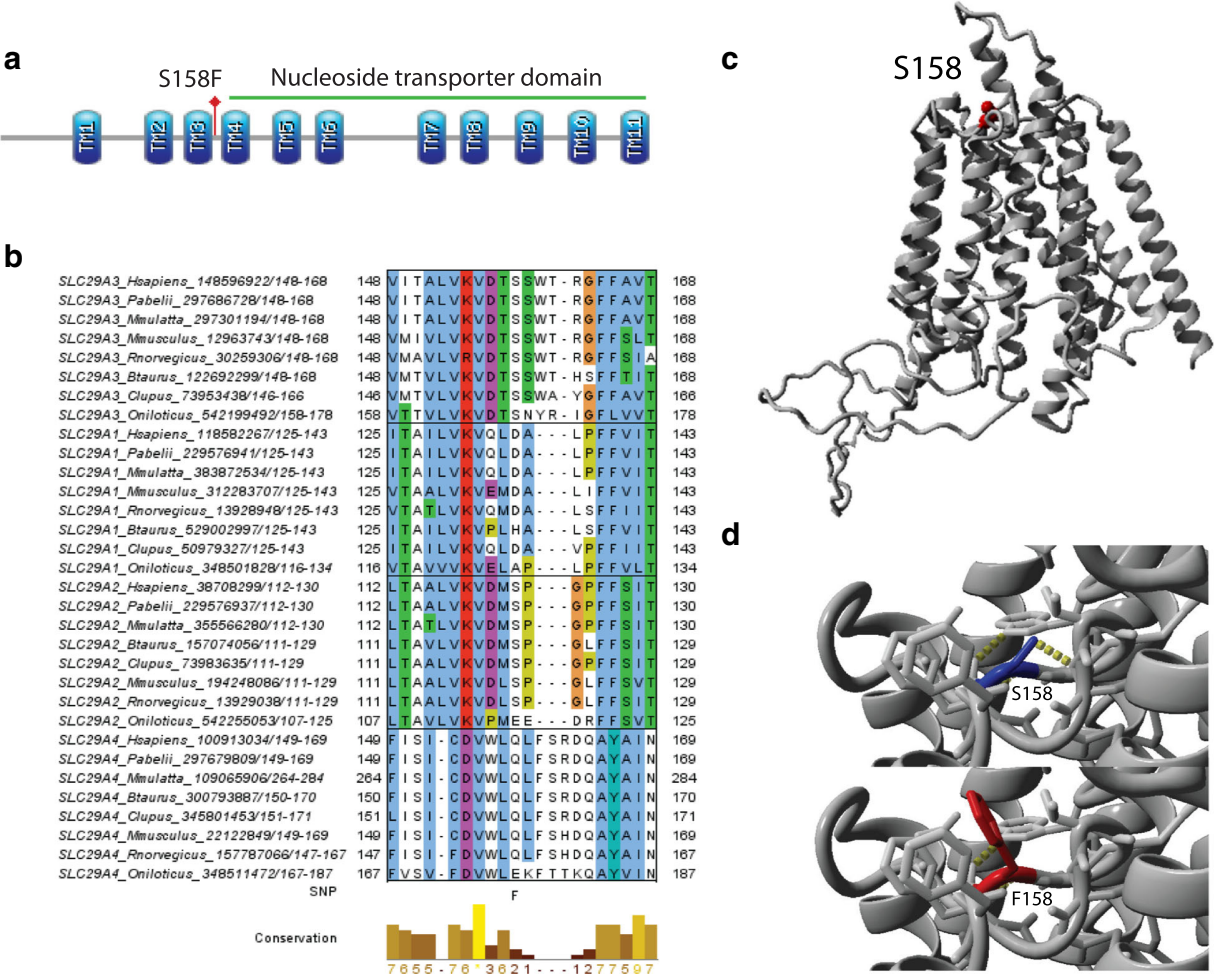
Result of detailed SNP analysis of SLC29A3 S158F. **a** Domain architecture of SLC29A3. The protein has 11 transmembrane helices and SLC29A3 S158F (red lollipop) lies between TM3 and TM4 which is localized extracellularly. Residues 169-473 correspond to the nucleoside transporter domain (Pfam:PF01733). **b** Multiple alignment using all four members in the SLC29 gene family i.e. SLC29A1-4. Orthologs of each member were retrieved using Orthologue search on ANNOTATOR [25]. MAFFT with L-INS-i was used to create the multiple alignment. **c** Homology model of SLC29A3 created by Memoir based on the template of a crystal structure of the glycerol-3-phosphate transporter from *E. coli* (PDB ID: 1PW4 chain A). This template was retrieved using HHPRED against PDB on ANNOTATOR [e-value = 1.7e-08]. **d** Screen shot of the FoldX result showing wild type and after mutation. The SNP was predicted to be stabilizing on protein structure (average ddG run over 5 times = − 1.14 kcal/mol SD = 0.04)

Our study also showed for the first time that patients who were carrying either the CT or TT of SLC29A3 473 C > T (rs780668) were associated with increased OS. SLC29A3 belongs to the equilibrative nucleoside transporter (ENT) family, responsible for passive nucleoside transport and has 11 transmembrane helices (TMs) within the nucleoside transporter domain (Pfam:PF01733) (Fig. 5a). SLC29A3 S158 is likely to be a subfamily specific residue since serine at this position is fully conserved from human to fish among SLC29A3 orthologs but cannot be seen in other members in the family (Fig. 5b). This residue may relate to its unique function compared to other members in that it seems to function in the inner membrane of mitochondria and/or in the lysosome which requires an acidic pH environment and the position of the SNP seems to localize outside of the inner membrane of mitochondria [39]. For homology modeling of SLC29A3, we had a problem retrieving correct TMs models using MODELLER with loop refinement. The template used was a crystal structure of the glycerol-3-phosphate transporter from *E.Coli* (PDB:1PW4) chain A which has only 12% identity to our query. This problem is common when we used MODELLER which is more suitable for modeling soluble proteins than membrane proteins. Therefore, we used another software called Memoir [40] which is a homology modelling algorithm designed specifically for membrane proteins. A homology model from this software retrieved 11 TMs with long N-terminus and long loop regions between TM6 and 7 and the correct SNP’s position on the 3D structure (Fig. 5c). According to FoldX, SLC29A3 S158F was predicted to have a significant destabilizing effect with average ddG of 2.18 kcal/mol which could be explained by the strong change from the polar side chain serine to the larger and more hydrophobic side chain phenylalanine (Fig. 5d). Besides potential involvement of the conserved wildtype serine in the transport process, increasing the hydrophobicity through the mutation at the outside interface with the membrane may result in deeper insertion of the affected helix in the membrane. SLC29A3 can transport gemcitabine into organelles, e.g., mitochondria [39]. Moreover, SLC29A3 could be involved in the mitochondrial toxicity of nucleoside drugs [8]. Since SLC29A3 S158F is most likely to affect protein function, it may have a significant impact on transporting gemcitabine into mitochondria.

**Fig. 6:**
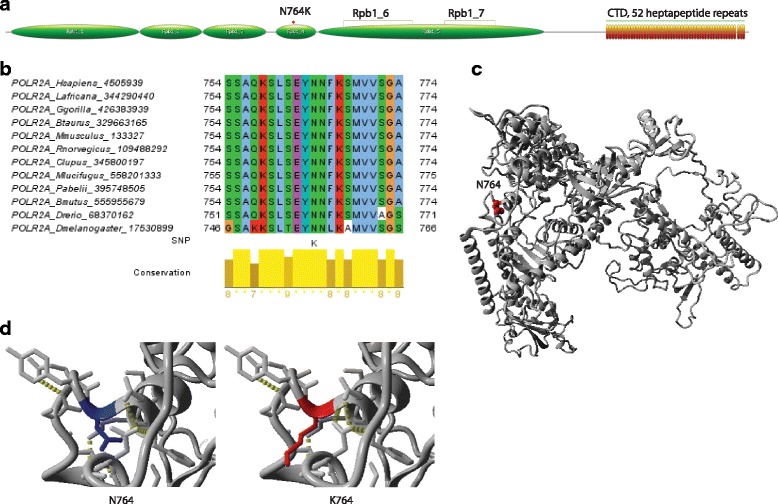
Result of detailed SNP analysis of POLR2A N764K. **a** Domain architecture of POLR2A. **b** Multiple alignment of POLR2A. Orthologs of POLR2A were retrieved using Orthologue search on ANNOTATOR [25]. MAFFT with L-INS-i was used to create the multiple alignment. **c** Homology model of POLR2A was created by MODELLER with loop refinement using a crystal structure of yeast RNA polymerase II (PDB:1I3Q chain A) as a template. This template was retrieved using BLAST against PDB on ANNOTATOR [e-value = 0.0]. **d** Screen shot of the FoldX result showing wild type and after mutation. The SNP was predicted to destabilize protein structure (average ddG run over 5 times = 1.13 kcal/mol, SD = 0.09)

POLR2A (DNA-directed RNA polymerase II subunit RPB1) encodes the largest subunit (out of 12 subunits) of RNA polymerase II (Pol II) which catalyzes the RNA synthesis from DNA. POLR2A contains a carboxy terminal domain (CTD) which is composed of 52 heptapeptide repeats that are necessary for the polymerase activity (Fig. 6a). POLR2A N764K is located in the RNA polymerase’s domain 4 (Pfam:PF05000) which is also known as the funnel domain. The N764 is highly conserved among orthologs (Fig. 6b) and both PolyPhen-2 and SIFT analyses predicted that the SNP affects protein function (Additional file 1: Table S2). We created a homology model of POLR2A without the CTD using a crystal structure of yeast RNA polymerase II (PDB:1I3Q chain A) as a template (%identity = 50.3%) (Fig. 6c). The SNP is in the loop region and it was predicted to have a destabilizing effect by FoldX (average ddG 1.13 kcal/mol) (Fig. 6d). This could be due to the longer and charged lysine side chain causing a change in the conformation of the local loop structure. In our study, interestingly, wild type (CC) is found to be associated with higher grade 3 or 4 thrombocytopenia when compared to the CT variant (Table 3). Further analysis is needed to understand the mechanism for this. There is an evidence that dFdCTP is incorporated into RNA which is concentration- and time-dependent, resulting in inhibition of RNA synthesis [41]. In human parental NSCLC cells with a different inherent gemcitabine resistance, sensitivity to gemcitabine was related to differences in RNA corporation [42]. Since the SNP is found to be strongly deleterious from our analyses, it would be interesting to investigate whether POLR2A itself plays a role in the gemcitabine pharmacologic pathway and whether POLR2A N764K SNP has any implications on RNA synthesis.

**Fig. 7:**
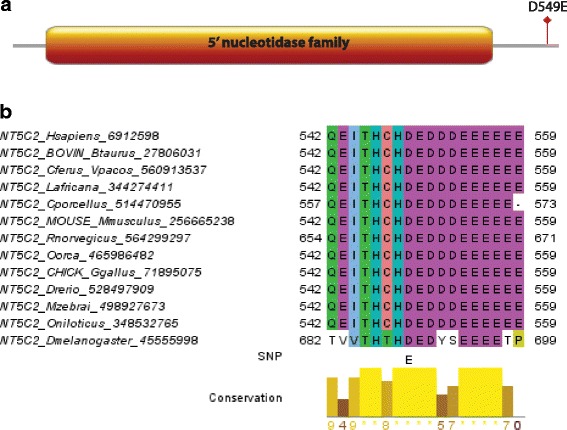
Result of detailed SNP analysis of NT5C2 D549E**. a** Domain architecture of NT5C2. **b** Multiple alignment of NT5C2. Orthologs of NT5C2 were retrieved using Orthologue search on ANNOTATOR [25]. MAFFT with L-INS-i was used to create the multiple alignment

NT5C2 (5′-nucleotidase, cytosolic II) encodes a hydrolase that serves a crucial role in cellular purine metabolism by acting primarily on inosine 5′-monophosphate (IMP) or guanosine monophosphate (GMP). The 5′-nucleotidase is a huge family of enzymes that catalyze the dephosphorylation of deoxy- and ribonucleoside monosphosphates to nucleoside analogues and inorganic phosphates [43, 44]. NT5C2 is known to dephosphorylate monophosphorylated gemcitabine. The NT5C2 D549 is the first charged amino acid in the last 13 acidic residues on the C-terminus of the protein (Fig. 7a). A study in 1999 proved the importance of the highly acidic C-terminus in NT5C2 protein using cDNA constructs encoding proteins lacking either N- or C-terminus and obtained the kinetic and molecular characteristics of the recombinant proteins [45]. When the last 13 acidic residues on the C-terminus were eliminated, there was a drastic reduction in the catalytic competence of the enzyme by lowering both the substrate affinity and the specific productivity. Furthermore, the capability of the protein to form a tetramer was significantly compromised. From the experiment above, it was concluded that the region of glutamic and aspartic acid residues in the C-terminus of the enzyme is necessary for the complete function of NT5C2. Another study, on bovine NT5C2 found that the C-terminus is perhaps involved with the modulation of enzyme function [46]. In our detailed SNP analysis, the D549 is well conserved among orthologs of NT5C2 (Fig. 7b) but is not conserved throughout the NT5C enzyme family (data not shown). D549E was predicted as deleterious by both PolyPhen-2 and SIFT. However, D and E are both negatively charged amino acids and the following acidic C-terminus shows a mixed pattern of the two so it is not mechanistically clear how this mutation could alter the enzyme activity of NT5C2. In agreement with this, we do not find significant correlations with survival or toxicity for NT5C2 D549E.

HELB T980I and CTDP1 T221M, unlike the other four SNPs that we predicted to directly affect the gemcitabine pharmacologic pathway, were selected because they could potentially affect the pathway indirectly in order to establish if more remotely related SNPs are still useful candidates for further studies. According to our criteria, HELB T980I passed three criteria i.e. significant results in SIFT, PolyPhen-2 and high MAF in Singaporean Chinese (Additional file 1: Table S2) while CTDP1 T221M passed two criteria (borderline) i.e. the SNP is in a domain region and has significant FoldX stability change (Additional file 1: Table S2). HELB T980I lies close to the phosphorylation sites in the phosphorylation regulated subcellular localization (PSLD) domain at the C-terminus of the protein. The PSLD domain has been suggested to play a significant role in regulating the subcellular localization of HELB [47]. The same study also suggested a possible role of HELB in DNA repair. Another study gives further evidence that HELB is recruited by Replication Protein A to mitigate replication stress [48]. So, we hypothesized that the SNP could impair HELB’s DNA replication stress mitigating effect of gemcitabine-induced DNA damage which could lead to reduced recovery from gemcitabine-induced replication stress, and hence to a better anti-cancer activity of gemcitabine. CTDP1 dephosphorylates a phosphorylated C-terminal domain of POLR2A to facilitate Pol II recycling for transcription [49]. There is evidence suggesting that CTDP1 may play a role in DNA damage response as well [50]. However, from our clinical results, we could not see any correlation between these two SNPs and the patient outcome. Therefore, it can be suggested that when we choose SNPs in the final step, we should expect a high chance for correlation with drug response only for those that are affecting the pharmacologic pathway directly.

## Conclusions

Overall, our bioinformatics approach can be used to select a small number of potential causative SNPs that can be rationalized with molecular mechanisms of their effects. Our workflow can also be applied to any pathway of interest that could affect other phenotypes. Nowadays, using GWAS to find SNPs associated with common diseases or adverse drug reactions are the norm but even though thousands of case samples have been used, one review found that about 30% of the results lead to a null finding [51]. Furthermore, significant GWAS hits are often not easily explainable to have functional effects e.g. when they are intronic or synonymous SNPs. Although our study may be limited in sample size, we managed to find that three out of six final candidate SNPs are associated with patient outcome. However, more association studies and possibly molecular and cellular studies are needed to further establish the value of these three SNPs as biomarkers of patient outcome. While we have to acknowledge that this approach of selective filters does not guarantee to find all SNPs involved in a studied phenotype, the main benefit is that one can reduce the space of possible candidates to a small experimentally tractable number with higher chance of being relevant. We do believe that other candidate SNPs that passed three out of five criteria would also be potential candidates to be tested further. We hope our approach and findings will pave the way to more meaningful biomarkers and personalized treatment options in the future.

## Additional file

Additional file 1: **Table S1.** Pyrosequencing primers of the six final candidate SNPs. **Table S2**. Detailed result of the 77 nsSNPs (in separated Excel file). **Table S3**. Genotyping result of the six final candidate SNPs for 90 NSCLC patient samples. (37 zip)

## Abbreviations

CI: Confidence Interval
ddG: Free energy change
dFdCTP: Gemcitabine triphosphate
MAF: Minor allele frequency
NSCLC: Non-small cell lung cancer
nsSNPs: non-synonymous SNPs
OS: Overall survival
PFS: Progression free survival
SNPs: Single Nucleotide Polymorphisms
