# Supplementary material for: Discovering novel SNPs that are correlated with patient outcome in a Singaporean cancer patient cohort treated with gemcitabine-based chemotherapy
Source: BMC Cancer. 2018 May 11;18:555. doi: 10.1186/s12885-018-4471-x (PMC5948914; doi:10.1186/s12885-018-4471-x)
Supplement: Supplementary file 1 — Table S1. Pyrosequencing primers of the six final candidate SNPs. Table S2. Detailed result of the 77 nsSNPs (in separated Excel file). Table S3. Genotyping result of the six final candidate SNPs for 90 NSCLC patient samples. (37 zip) [file 12885_2018_4471_MOESM1_ESM.zip › Supplement_info_2018_finalR2.docx]

**Supplementary Information**

**Supplementary Tables**

**Table S1** Pyrosequencing primers of the six final candidate SNPs **2**

**Table S2** Detailed result of the 77 nsSNPs (in separated Table)

**Table S3** Genotyping result of the six final candidate SNPs for **90 NSCLC** patient samples **3**

**Table S1 Pyrosequencing primers of the six final candidate SNPs**

| **Official gene symbol** | **SNP (rsid)** | **Forward** | **Reverse** | **Sequencing** | **PCR product size (bp)** |
| --- | --- | --- | --- | --- | --- |
| ABCG2 | rs2231142 | *biot*/actgcaggttcatcattagctaga | ccgttcgtttttttcatgattc | cgaagagctgctgagaa | 238 |
| SLC29A3 | rs780668 | *biot*/ctggccatcttcatggtgataact | atatcagtgcctgggagttcct | agccacgggtccagg | 181 |
| NT5C2 | rs3740387 | ctacaagcggcaccagctga | *biot/*acctcgtttgttcctgtgagtcc | ggaaattacacactgcca | 201 |
| POLR2A | rs2228130 | atcaggtgaaccgcattctt | *biot*/acctgggagatgttaatcttgg | atccctgtctgaatacaa | 136 |
| HELB | rs1168312 | *biot*/tcagctccggaatgccatta | aattgccatctttcagcaaaggt | ggagtggagatgctgac | 267 |
| CTDP1 | rs2279103 | aaatctctttccttgtggagactc | *biot*/agaatcaggaaggaggttaccc | gtcccgagaatctcag | 191 |

*biot*/ - primer is biotinylated at the 5’ end.

**Table S3 Genotyping result of the six final candidate SNPs for 90 NSCLC patient samples**

| **Genotype** | **No. of Patients** |
| --- | --- |
| **ABCG2 Q141K (rs2231142, c.421 C>A)** |  |
| CC | 48 |
| CA | 36 |
| AA | 6 |
| **SLC29A3 S158F (rs780668, c.473C>T)** |  |
| CC | 23 |
| CT | 46 |
| TT | 21 |
| **NT5C2 D549E (rs3740387, c.1647C>T)^+^** |  |
| CC | 21 |
| CT | 50 |
| TT | 19 |
| **POLR2A N764K (rs2228130, c.2292C>T)** |  |
| CC | 80 |
| CT | 10 |
| TT | 0 |
| **HELB T980I (rs1168312, c.2939C>T)*** |  |
| CC | 50 |
| CT | 32 |
| TT | 6 |
| **CTDP1 T221M (rs2279103, c.662C>T)** |  |
| CC | 68 |
| CT | 20 |
| TT | 2 |

**^+^**Latest dbSNP build annotated this SNP as NT5C2 D549D (synonymous snp).

**^*^**Genotype could not retrieve from 2 out of 90 patients for the HELB T980I
